# Evaluation of gaps in direct oral anticoagulants (DOACs) management in an outpatient underserved clinic: a cross-sectional study

**DOI:** 10.1038/s41598-025-07712-0

**Published:** 2025-07-02

**Authors:** Bader M. Alghamdi, Sara L. Rogers

**Affiliations:** 1 Department of Clinical Pharmacy, College of Pharmacy, Al-Baha University, Al-Baha, Saudi Arabia; 2 Department of Pharmacy Practice, Texas A&M Irma Lerma Rangel College of Pharmacy, Kingsville, TX USA; 3 American Society of Pharmacovigilance, Houston, TX 77225 USA

**Keywords:** Anticoagulant agent, Outpatient care, Quality improvement, Outcomes research, Acute coronary syndromes, Heart failure

## Abstract

DOACs are widely used for stroke prevention in non-valvular atrial fibrillation and treatment of thromboembolism, but gaps in their management can lead to adverse outcomes. In outpatient, underserved clinics, challenges such as inappropriate dosing, lack of monitoring, and improper drug combinations may be more pronounced, necessitating evaluation to optimize patient care and reduce risks. This study assessed the appropriateness of DOAC use and dosing per FDA guidelines, considering renal function, hepatic function, and drug–drug interactions. Secondary objectives focused on evaluating inappropriate aspirin use alongside DOACs. This was a single-center retrospective cohort study. The study was conducted at Texas A&M Health Family Care Clinic (12/24/2022–12/24/2023). Older than 18 years of age and had active prescription of DOAC (apixaban, dabigatran, edoxaban, or rivaroxaban). A total of 125 DOAC patients were included. Inappropriate dosing based on renal function occurred in 16% of cases. DOAC use was unsuitable in two patients with severe hepatic impairment. Major drug interactions resulted in two instances of inappropriate DOAC use. Additionally, about 61% of aspirin usage involved inappropriate combinations with DOACs, as shown in Fig. 1. The study’s findings indicate that anticoagulation management in our ambulatory care setting has the potential for further optimization.

## Introduction

DOACs offer several advantages over traditional anticoagulants like warfarin, making them increasingly popular in clinical practice. However, despite these advantages, DOACs are not without risks. Patients may still experience complications, such as bleeding events, particularly in populations with specific comorbidities or in those taking multiple medications^1^.

The study from 2017 to 2019 revealed that anticoagulants, including DOACs, were significant contributors to emergency department visits due to adverse drug events, particularly among older adults at higher risk for complications^2^. A notable proportion of these visits was linked to bleeding issues associated with anticoagulant therapy, highlighting the need for better monitoring and management strategies to enhance patient safety. Root cause analyses of outpatient anticoagulation management often highlight key issues such as inadequate patient education, poor renal and hepatic function monitoring, and ineffective management of drug interactions. These gaps can result in inappropriate dosing and adverse events^3^.

Patients and providers in rural and underserved areas may benefit from using DOACs over warfarin due to their ease of use and lack of routine INR monitoring, important aspects of DOAC monitoring are often overlooked in these settings^4^. As a result, rural patients may face a higher risk of inappropriate dosing and insufficient monitoring of renal function and drug interactions, both critical for safe DOAC use. Structured education for primary care providers and establishing better monitoring systems are essential for improving anticoagulation management^5,6^.

The first step in developing an anticoagulation stewardship model involves conducting a gap analysis, which assesses current practices related to patient education, renal and hepatic function monitoring, and drug interaction management. This analysis helps identify deficiencies and allows healthcare providers to improve DOAC use, ensuring safe and effective anticoagulation management aligned with FDA recommendations and best practices. This approach can lead to improved patient outcomes, particularly in underserved rural settings^7^.

## Methodology

### Study design

This was a cross-sectional retrospective analysis of patients on DOACs in an outpatient clinic setting. Patients who had an office visit at Texas A&M Family Care Clinic between December 24, 2022, and December 24, 2023, and who had a DOAC listed on their home medication profile were identified through a query of clinic records. Patients were included in the analysis if they were older than 18 years of age and had been prescribed a DOAC (apixaban, dabigatran, edoxaban, or rivaroxaban). Patients were excluded if they were 18 or younger or did not have clinic office visits during active DOAC therapy. This study was approved by the Institutional Review Board of the study institution. All methods were performed in accordance with the relevant guidelines and regulations.

**Fig. 1:**
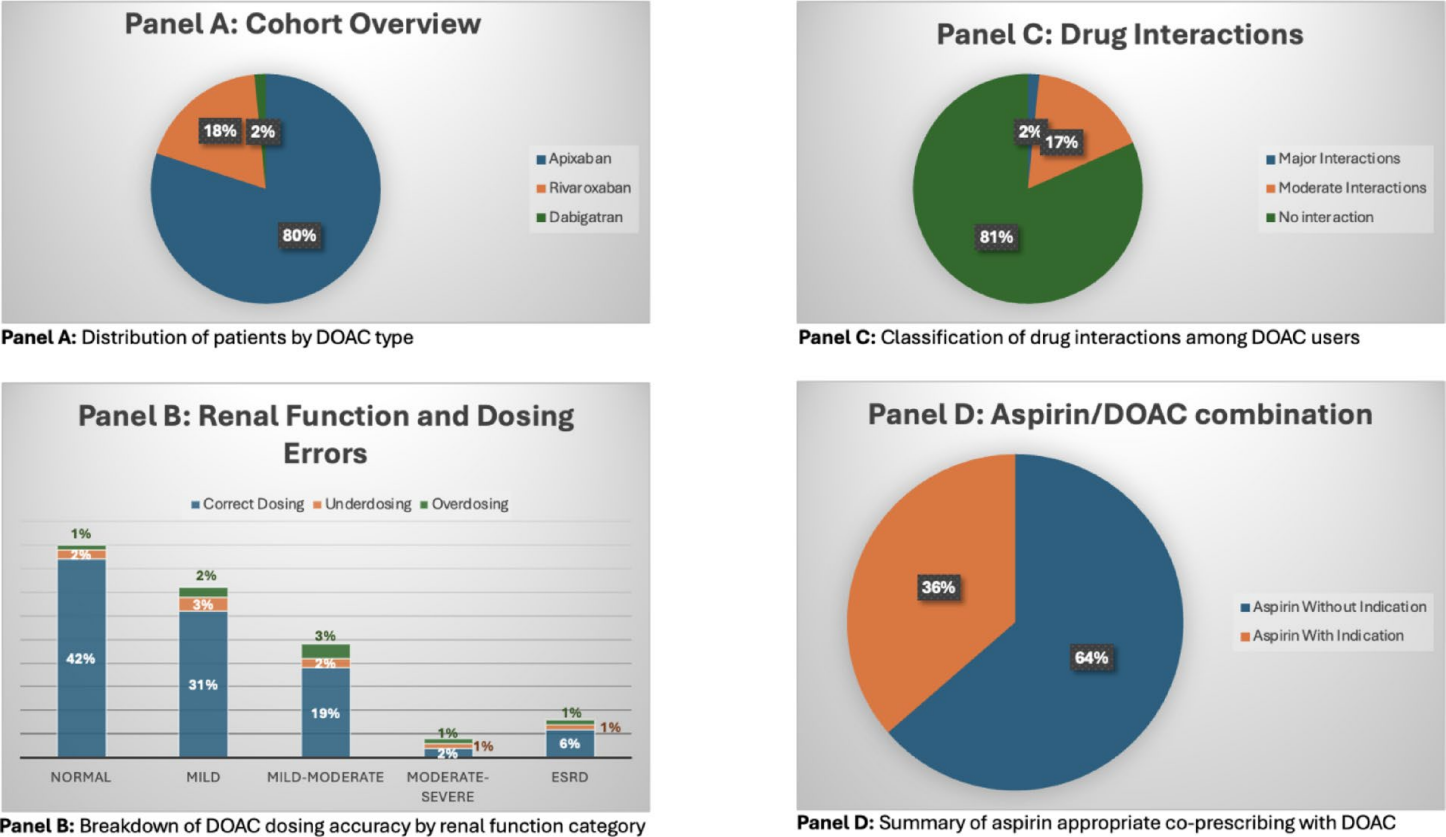
DOAC prescribing patterns in a cohort of 125 patients.

### Data collection

Data were collected by the fourth-year pharmacy interns’ team members using a data collection form. A data collection standard of operation was provided, describing the data collection steps from the charts to ensure that a unified and accurate collection is performed. The de-identified data were entered into a password-protected Microsoft Office Excel version (16.89.1) spreadsheet maintained on a secure server. An ambulatory care pharmacy resident conducted random audits to provide quality assurance. Baseline patient characteristics collected included age, gender, role of the physician in the patient’s care, relevant past medical history, height, and weight. Information related to DOAC use was collected, including most recently prescribed DOAC, anticoagulation indication, dose, date of initiation, and prescriber. Additional information collected included the date of the office visit, monitoring parameters, prior anticoagulant use, concomitant medications, and laboratory data to evaluate renal function, hepatic function, and hemoglobin/hematocrit from the most recent DOAC prescription, on or before the most recent Primary Care Physician (PCP) office. If a patient had a history of use of multiple DOACs, the most recent DOAC prescribed was utilized. If multiple indications for anticoagulant use were possible, physician documentation was reviewed to assess the current indication for anticoagulation. If the most recent or previously used anticoagulant had been discontinued, the reason for discontinuation was assessed by manual review of physician documentation. Documentation of monitoring for renal and hepatic function. Creatinine clearance (CrCl) was calculated utilizing the Cockcroft–Gault equation. Actual body weight was used.

### Objectives

The primary objective was to evaluate whether DOAC use and dosing were appropriate based on FDA-labeled recommendations, considering renal function, hepatic function, and drug–drug interactions. The secondary objectives centered on examining the inappropriate use of aspirin in combination with DOACs within our patient cohort.

### Analysis

Dosing was evaluated based on currently available FDA-approved package labeling from the package insert^8–10^. Renal function was assessed based on serum creatinine and CrCl. Renal function was categorized based on estimated creatinine clearance (CrCl) calculated using the Cockcroft–Gault equation. The following classifications were used: normal renal function (CrCl ≥ 90 mL/min), mild impairment (CrCl 60–89 mL/min), moderate impairment (CrCl 30–59 mL/min), severe impairment (CrCl 15–29 mL/min), and kidney failure (CrCl < 15 mL/min or on dialysis). This stratification is commonly used in pharmacokinetic studies to guide medication dosing^11^. Other factors such as age and weight for apixaban have been considered. For apixaban, dose reduction to 2.5 mg twice daily was considered appropriate if at least two of the following criteria were met: age ≥ 80 years, body weight ≤ 60 kg, or serum creatinine ≥ 1.5 mg/dL. For rivaroxaban, the standard dose of 20 mg once daily was used for patients with CrCl ≥ 50 mL/min; a reduced dose of 15 mg once daily was appropriate for CrCl 15–49 mL/min. DOACs were considered contraindicated in patients with CrCl < 15 mL/min or those on dialysis unless otherwise stated in specific labeling. Dabigatran dose reductions were evaluated based on CrCl thresholds (e.g., 150 mg BID for CrCl > 30 mL/min, and 75 mg BID for CrCl 15–30 mL/min)^8–10^.

Hepatic function was evaluated using Child–Pugh scores considering the following data for calculating Child–Pugh scores: bilirubin, albumin, alkaline phosphatase, AST, ALT, and INR if available.

Drug–drug interaction significance was determined based on pharmacologic mechanisms and clinical management guidance provided in the DOAC Playbook, Anticoagulation Forum^12^. Interactions were categorized as either pharmacodynamic (e.g., increased bleeding risk with NSAIDs, SSRIs, antiplatelets) or pharmacokinetic (e.g., increased or decreased DOAC concentrations via P-gp and/or CYP3A4 inhibition/induction). Interactions were considered clinically significant if they warranted dose adjustment, avoidance, or enhanced monitoring according to the Playbook^8–10^.

For the aspirin, we analyze the appropriate combination of DOAC and aspirin based on the indication listed in the aspirin FDA-approved package labeling and the current clinical guideline^13,14^. Aspirin was the primary antiplatelet assessed due to its higher prevalence of inappropriate use with anticoagulants, likely related to its over-the-counter availability and perception as low-risk.

For each patient, the appropriateness of DOAC dosing was determined based on the FDA-approved package insert for the documented indication. When a valid indication was present [e.g., treatment of VTE or stroke prevention in non-valvular atrial fibrillation (NVAF)], the prescribed dose was compared against the recommended dose for that indication. If no indication was documented or if the DOAC was prescribed for a non-approved indication, this was classified as potentially inappropriate use.

All data including concomitant medications, medical history, and physician notes were taken from the index visit. The index visit is the visit associated with prescribing or refilling the DOAC and it was found on the same day of the DOAC prescribing and has physician information on anticoagulation prescription. The most recent lab results ordered during the index office visit were used for evaluation. In evaluating the documentation of monitoring laboratory tests, tests were considered appropriately documented if they had been obtained within 1 year of the index visit. Microsoft Office Excel version (16.89.1) was used for all statistical analysis.

## Results

125 patients were identified for possible inclusion in the analysis, with a mean age of 70 years—100 apixaban, two dabigatran, and 23 rivaroxaban (Fig.1, panel A). No patients have been prescribed edoxaban. The cohort consisted of 44.8% males and 55.2% females. The most common indication for anticoagulation was stroke prevention with NVAF (64.8%). The cases of patients were all handled by family medicine physicians or their residents. There were 12 new prescriptions, accounting for 9.6% of the total, and 113 refills, making up 90.4% of the total. Full baseline patient characteristics can be found in Table 1.

**Table 1:** Baseline patient characteristics.

Characteristic	All DOACs (n = 125)	Apixaban (n = 100)	Rivaroxaban (n = 23)	Dabigatran (n = 2)
Age—years—mean ± SD	70 ± 13	70 ± 13	73 ± 11	75 ± 1
Gender—n (%)				
Female	69 (55.2%)	47 (47%)	9 (39%)	–
Male	56 (44.8%)	53 (53%)	14 (61%)	2 (100%)
Weight—kg—Mean ± SD	96 ± 33	94 ± 34	99 ± 32	92 ± 31
BMI—kg/m^2^—mean ± SD^a^	34 ± 10	34 ± 12	34 ± 11	32 ± 14
< 18.5—n (%)	3 (2.4%)	3 (3%)	–	–
18.5 and 24.9—n (%)	19 (15.2%)	15 (15%)	3 (13%)	1 (50%)
25–29.9—n (%)	25 (20.8%)	20 (20%)	5 (21.7%)	–
≥ 30—n (%)	74 (59.2%)	59 (59%)	14 (60.8%)	1 (50%)
Indication—n (%)				
Prevention of VTE&PE	25 (20.0%)	19 (19%)	6 (26%)	–
Treatment of VTE&PE	9 (7.2%)	8 (8%)	1 (4.3%)	–
Prevention of Stroke in NVAF	81 (64.8%)	65 (65%)	15 (65%)	1 (50%)
Coronary artery disease	4 (3.2%)	3 (3%)	–	1 (50%)
Other diagnoses	6 (4.8%)	5 (5%)	1 (4.3%)	–
Concurrent medication use—n (%)				
Aspirin	33 (26.4%)	28 (28%)	4 (17.3%)	1 (50%)
Clopidogrel^b^	6 (5.6%)	6 (6%)	–	1 (50%)
Dual antiplatelet	3 (2.4)	2 (2%)	–	1 (50%)
NSAID	10 (8%)	10 (10%)	–	–
SSRI/SNRI	9 (7.1%)	6 (6%)	3 (13%)	–
Medical history—n (%)				
Hypertension	97 (77.6%)	73 (73%)	22 (95.6%)	2 (100%)
Dyslipidemia	53 (42.4%)	38 (38%)	15 (65.2%)	–
Diabetes mellites	51 (40.8%)	38 (38%)	12 (52%)	1 (50%)
Congestive heart failure	42 (33.6%)	35 (35%)	6 (26%)	1 (50%)
History of myocardial infarction	8 (6.4%)	7 (7%)	–	1 (50%)
History of GI bleed	3 (2.4%)	3 (3%)	–	–
CHADS2 score—mean ± SD	2.16 ± 1	2.07 ± 1	2.47 ± 1	3 ± 2
HAS-BLED score—mean ± SD	1.48 ± 1	1.47 ± 1	1.47 ± 1	2 ± 1

*BMI* body mass index, *CrCl* creatinine clearance, *SD* standard deviation, *Wt* weight, CHADS2, *CHF history* hypertension history, Age ≥ 75 years, Diabetes mellitus history, Stroke or TIA symptoms previously; HAS-BLED, Hypertension, Renal disease, Liver disease, Stroke history, Prior major bleeding or predisposition to bleeding, Labile INR, Age > 65, Medication usage predisposing to bleeding, Alcohol use.

^a^BMI values were calculated only for patients with available weight and height data (n = 122).

^b^Other antiplatelets such as ticagrelor and prasugrel were not observed in our study population.

In our cohort, 42% of patients had normal renal function, 31% had mild impairment, 19% had mild to moderate impairment, 2% had moderate to severe impairment, and 6% were classified as having end-stage renal disease (ESRD). Among the patients evaluated, 16% received DOAC doses that did not align with FDA-recommended dosing based on their renal function. Specifically, there were 9 cases of underdosing and 8 cases of overdosing (Fig.1, panel B).

For apixaban, there were 13 cases of inappropriate dosing: six cases of underdosing where patients were given a reduced dose without meeting at least two of the three criteria required for dose reduction, and seven cases of overdosing where patients received a higher dose despite meeting two of the three criteria for a dose reduction. For rivaroxaban, there were four cases of inappropriate dosing: three cases of underdosing without any clear justification although had a normal renal function, and one case of overdosing, particularly in patients with ESRD, where a lower dose should have been prescribed.

Among patients with hepatic impairment, 97% had mild impairment (Child–Pugh A), 2% had moderate impairment (Child–Pugh B), and 1% had severe impairment (Child–Pugh C). Of those assessed, there were 2 cases where DOACs were used: one involving apixaban and one involving rivaroxaban, both in patients with severe impairment (Child–Pugh C). According to FDA guidelines, alternative anticoagulation should be considered for these patients based on their hepatic function.

For the drug–drug interactions among the identified interactions, two cases involved major interactions with strong CYP3A4 and P-gp inhibitors or inducers, leading to the need for dose reductions or the avoidance of therapy. However, there were 21 cases where patients on DOAC were concurrently taking dual P-gp and moderate CYP3A4 inhibitors. While these cases generally did not require dose reductions, careful monitoring for signs of bleeding was to be recommended (Fig.1, panel C). Common clinically relevant drug interactions with DOACs are found in Table 2.

**Table 2:** Common clinically relevant drug interactions with DOACs.

Interacting drug/class	Type of interaction	Clinical significance (per DOAC playbook)
Antiplatelets (e.g., aspirin, clopidogrel)	Pharmacodynamic—↑ bleeding risk	Avoid or assess risk/benefit
NSAIDs (e.g., ibuprofen, naproxen)	Pharmacodynamic—↑ bleeding risk	Avoid or assess risk/benefit
SSRIs/SNRIs	Pharmacodynamic—↑ bleeding risk	Assess bleeding risk, not always contraindicated
P-gp Inducers (e.g., rifampin, phenytoin, carbamazepine)	Pharmacokinetic—↓ DOAC level → ↑ thrombotic risk	Avoid use
P-gp Inhibitors (e.g., amiodarone, verapamil, diltiazem)	Pharmacokinetic—↑ DOAC level → ↑ bleeding risk	Dose adjust or avoid depending on renal function
Strong CYP3A4 Inducers (e.g., St. John’s Wort, rifampin)	Pharmacokinetic—↓ DOAC level → ↑ thrombotic risk	Avoid use
Strong CYP3A4 Inhibitors (e.g., ketoconazole, ritonavir)	Pharmacokinetic—↑ DOAC level → ↑ bleeding risk	Avoid use or adjust dose
Combined P-gp + CYP3A4 Inhibitors (e.g., clarithromycin, itraconazole)	Pharmacokinetic—↑ DOAC level → ↑ bleeding risk	Avoid use or adjust dose

Out of 33 patients, 21 were taking both DOACs and aspirin without a documented FDA-approved indication for aspirin use. Among the 12 patients with valid indications for aspirin, seven were using it for secondary prevention of ischemic stroke, four for coronary artery disease (CAD), and one for coronary stenting (Fig.1, panel D). However, per recent guidelines from the American Heart Association, the combination of DOACs and antiplatelets is generally discouraged in patients with stable CAD beyond 12 months post-stenting. Therefore, some of these cases—particularly those with stable CAD—may still represent potentially inappropriate use^14^.

Overall, dosing accuracy was highest in patients with normal renal function and mild to moderate hepatic function. However, dosing errors were more frequent in patients with abnormal renal function and those over 75 years old. The patients with severe hepatic function didn’t get the right DOAC recommendation.

## Discussion

This study aimed to assess the prescribing patterns of DOACs in an underserved setting. Timely dose modifications are crucial to mitigating long-term bleeding and thrombotic risks. Our results underscore the importance of establishing a monitoring program to ensure optimal DOAC management in this patient population. These findings align with research from metropolitan areas, such as the study by Brigham and Women’s Hospital, which reported the need for DOAC dose adjustments in approximately 10% of their patient cohort—a rate similar to the inappropriate dosing identified in our institution^15^.

Moreover, primary care providers, especially in rural areas, are facing increased patient loads due to the growing prevalence of chronic conditions, contributing to physician burnout. In clinical practice, physicians often have limited time to address all aspects of DOAC maintenance and monitoring^6,7^. As shown in Table 1, nearly all patients in our study had two or more chronic conditions being managed during the same visit. The heavy workloads, extended working hours, and administrative burdens further contribute to the rising rates of physician burnout, particularly in rural and underserved areas where shortages in family care physicians are more pronounced. This situation underscores the critical need for a more structured, pharmacist-led monitoring approach to support DOAC therapy in such settings^15,16^.

Our finding that 16% of patients received inappropriate DOAC dosing is comparable to previous reports. For example, Steinberg et al. reported inappropriate dosing in approximately 13.3% of atrial fibrillation patients in a national registry, while Siontis et al. found a 15% rate of under- or overdosing among Medicare beneficiaries with NVAF. These similarities suggest that inappropriate DOAC dosing is a widespread issue, not limited to our rural underserved population, although differences in patient characteristics and provider type may contribute to some variability.

Despite the valuable insights gained from this study, several limitations must be considered. One limitation is that the study focused solely on dosing patterns, and due to its retrospective design, we were unable to assess important factors such as patient adherence, clinical outcomes like bleeding or thromboembolic events, or gaps in DOAC education. However, the development of a comprehensive monitoring program for DOAC management would need to address all these aspects, as incorporating them would likely result in improved outcomes in terms of the safe and effective use of DOACs, as supported by other research^17^. Furthermore, because this was a retrospective chart review, we could not assess whether deviations from guideline-recommended DOAC dosing were influenced by provider clinical judgment, shared decision-making with the patient, or undocumented patient preferences. Cases lacking rationale in the record were classified as inappropriate, although the true intent behind the prescribing decision could not always be determined.

Our study provides a foundational basis for the development of an anticoagulation stewardship model. Conducting a gap analysis is the initial step in establishing an effective anticoagulation stewardship program, and additional evidence on both prescribing patterns and clinical outcomes will be necessary to identify the optimal approach for creating a successful model tailored to specific healthcare settings^7^.

The rural setting provides a unique perspective on anticoagulation management, with challenges and opportunities that may vary from those in other healthcare environments. One notable challenge is the underappreciation of the need for improved DOAC management, stemming from a common misconception among both patients and providers that DOACs carry less risk than warfarin, which could result in unintended adverse outcomes^18^.

Although our study provides valuable insights into DOAC usage and prescribing practices, additional research is needed to enhance anticoagulation management strategies across various healthcare settings by incorporating newly developed DOAC monitoring models.

## Conclusion

Our study underscores the critical need for an anticoagulation stewardship program, particularly focusing on the maintenance monitoring of DOACs in outpatient settings. Our findings reveal a substantial number of patients receiving inappropriate DOAC doses, primarily due to renal and hepatic impairment, as well as unaddressed drug–drug interactions. The prevalent use of aspirin without a documented FDA-approved indication further complicates patient safety by increasing the risk of bleeding.

## Data Availability

The datasets generated and/or analyzed during the current study are not publicly available but are available from the corresponding author on reasonable request. Please contact Bader M Alghamdi at balghamdi@bu.edu.sa.
